# FasL (rs763110) gene polymorphism is not associated with susceptibility to rheumatoid arthritis in Croatian population

**DOI:** 10.3325/cmj.2020.61.547

**Published:** 2020-12

**Authors:** Marinko Artuković, Marina Ikić Matijašević, Antonio Markotić, Alan Šućur, Danka Grcevic, Nataša Kovačić, Darja Flegar, Asja Stipić Marković, Dino Šisl, Irena Artuković, Tomislav Kelava

**Affiliations:** 1 Department of Clinical Immunology and Pulmonology, Sveti Duh University Hospital, Zagreb, Croatia; 2 Center for Clinical Pharmacology University Clinical Hospital Mostar, Mostar, Bosnia and Herzegovina; 3 Department of Physiology and Immunology, University of Zagreb School of Medicine, Zagreb, Croatia; 4 Laboratory for Molecular Immunology, Croatian Institute for Brain Research, School of Medicine University of Zagreb, Zagreb, Croatia; 5 Polyclinic for the Prevention of Cardiovascular Diseases and Rehabilitation, Zagreb, Croatia

## Abstract

**Aim:**

To investigate the association of FasL gene polymorphism (rs763110) with rheumatoid arthritis occurrence, disease activity, and tumor necrosis factor-α (TNF-α) plasma concentration in Croatian patients, and to conduct an updated meta-analysis.

**Methods:**

This cross-sectional study enrolled 81 patients with rheumatoid arthritis and 94 control patients. After the assessment of the Disease Activity Score (DAS)-28, blood was taken for analysis. DNA was isolated from the whole blood to determine FasL polymorphism (rs763110) by polymerase chain reaction. Protein levels of TNF-α were determined with ELISA. After a detailed literature search, we conducted an updated meta-analysis using the Review Manager 5 software.

**Results:**

Rheumatoid arthritis patients had significantly higher TNF-α concentration in plasma (1.65 [1.2-2.42] pg/mL) than controls (0.99 [0.77-1.35] pg/mL, *P* < 0.001). The FasL rs763110 polymorphism was not associated with rheumatoid arthritis occurrence in either codominant, dominant, recessive, overdominant, or log additive model. Furthermore, the rs763110 genotype was not associated with DAS 28 score or TNF-α concentration. After we added our results to an updated meta-analysis, the significant association previously reported for Western Eurasians was abolished.

**Conclusion:**

Our data suggest that the association between FasL rs763110 polymorphism and RA susceptibility in Western Eurasians observed in previous studies might be overestimated and should be limited to the population of Southwestern Asia until further investigations are performed.

Rheumatoid arthritis (RA) is a chronic systemic autoimmune disease characterized by symmetric inflammation of synovial joints, which causes pain and stiffness, and eventually results in joint destruction and disability. The etiology of RA is multifactorial and not completely elucidated, but it is generally accepted that disease occurrence and progression are affected by both genetic and environmental factors. The major part of genetic susceptibility to RA can be ascribed to the presence of specific HLA genotypes, such as HLA-DR1, HLA-DR4, and HLA-DR10 (1,2).

However, the presence of specific HLA genotype cannot entirely explain the genetic risk for the development and severity of RA, so ongoing research investigates the association of RA with gene polymorphisms of various other genes involved in the function and regulation of the immune system. As dysregulated immune cells apoptosis plays a role in the pathogenesis of RA, such investigations often focus on genes coding the molecules involved in the regulation of apoptosis, such as FasL, Fas, Caspase 8, Death Receptor 3, and Bcl2 (3-5).

Numerous investigations have confirmed that the Fas/FasL apoptotic pathway plays an important role in the pathogenesis of RA. High Fas receptor levels are expressed on the synoviocytes of RA patients, and both Fas and FasL are expressed on synovial macrophages and T lymphocytes (6). Furthermore, Fas/FasL apoptotic pathway can be modulated by TNF-α, a pivotal cytokine in RA pathogenesis. Dysregulation of the Fas/FasL pathway in RA is evident by rarely observed apoptosis in the inflamed synovium, despite the mentioned increase in the synovial levels of FasL and Fas. Even though the activation of the Fas/FasL pathway was considered as a treatment for RA, data from *in-vitro* and *in-vivo* models have shown that Fas activation may stimulate inflammation rather than induce apoptosis (7-9). In addition, in response to Fas activation resting T cells proliferate while cycling cells enter apoptosis. Activation of the Fas receptor also depends on the form of FasL, as soluble FasL, which is increased in RA, induces synoviocyte proliferation and blocks the apoptotic effects of the membrane-bound FasL (10,11). Other than in RA, dysregulation of the Fas/FasL system has also been reported in various cancers, where it promotes tumor growth *in-vitro* and *in-vivo* (12).

Several polymorphisms have been implicated in the function of the Fas/FasL system, with the rs763110 (-844C>T) variant in the FasL gene emerging as an important research target. It has been shown that the rs763110 TT genotype suppresses apoptosis by reducing the binding affinity of FasL promoter for its target transcription factors (13). Even though individual studies have so far reported conflicting or inconclusive results regarding the effect of rs763110 on the disease risk, a couple of recent meta-analyses showed its possible protective effect regarding the risk of head and neck cancers, gynecological cancers, and post-radiotherapy toxic effects (14-16). On the other hand, meta-analyses by Zhu et al (5) and Lee et al (3) suggested that the presence of FasL rs763110 C/T polymorphism significantly increased the risk of RA in the Western Eurasian population (OR = 1.366, 95%CI = 1.093-1.707, *P* = 0.006). However, the Western Eurasian population in both meta-analyses was represented only by the Iranian and Turkish populations assessed in three individual studies. To the best of our knowledge, the association between RA and rs763110 C/T polymorphism was not studied in any European population. Therefore, in the present study, we investigated the association between rs763110 C/T polymorphism and the occurrence of RA in the Croatian population. We also aimed to determine if the rs763110 genotype was associated with disease activity as assessed by Disease Activity Score-28 (DAS 28) score and with the levels of TNF-α, the key cytokine in RA pathogenesis.

## Patients and methods

### Patients

After obtaining the approval from the Ethics Committee of the Sveti Duh University Hospital, the study enrolled 81 RA and 94 control patients. RA was diagnosed according to the criteria of the American College of Rheumatology (ACR/EULAR 2010), and RA patients were enrolled during the regular checkup at the Department of Clinical Immunology, Rheumatology and Pulmonology, Sveti Duh University Hospital from 2014 until 2017. Patients were recruited after having been diagnosed with RA at least two years before study enrollment, and if they had no previous periods of remission despite the use of persistent classical disease-modifying antirheumatic drugs (DMARD), nonsteroidal anti-inflammatory drugs, and/or corticosteroid therapy (no biological or targeted synthetic DMARDs). The control group consisted of age- and sex-matched patients without RA recruited at the same institution during the same period. Patients with acute or chronic inflammatory conditions and other rheumatic or autoimmune diseases were excluded from the study. After receiving written informed consent, 5 mL of venous blood was collected from each participant, and DAS 28 was determined in RA patients. Following sample collection, 250 μL of blood was frozen at -20 °C until DNA isolation (17). From the remaining sample, plasma and peripheral blood mononuclear cells were separated using Histopaque (Sigma-Aldrich, St. Louis, MO, USA). Plasma was collected and stored at -20 °C until analysis.

### Gene expression

Total RNA was extracted from peripheral blood mononuclear cells using TRIzol (Invitrogen by Life Technologies [LT], Grand Island, NY, USA), converted to complementary DNA and amplified in duplicates by qPCR in an ABI Prism 7500 Sequence Detection System (Applied Biosystems by LT). Gene expression of TNF-α and glyceraldehyde 3-phosphate dehydrogenase was assessed using TaqMan Assays (Applied Biosystems by LT) and presented as RNA relative quantity, as previously described (8).

### Extraction of DNA and genotyping of FasL single nucleotide polymorphism

Patients’ DNA was extracted from peripheral blood using the PureLink Genomic DNA kit (Invitrogen by Thermo Fisher Scientific, Waltham, MA, USA) according to the manufacturer's instructions. FasL rs763110 C/T polymorphism was genotyped on an ABI Prism 7500 Sequence Detection System (Applied Biosystems) using the TaqMan SNP Genotyping Assay (Assay ID: C_3175437_10).

### Determination of TNF-α concentration in plasma

Concentrations of TNF-α in plasma were determined with ELISA using the commercially available kits (Quantikine Immunoassay, R&D systems, Minneapolis, MN, USA) according to the manufacturer's instructions.

### Updated meta-analysis

**Figure 1:**
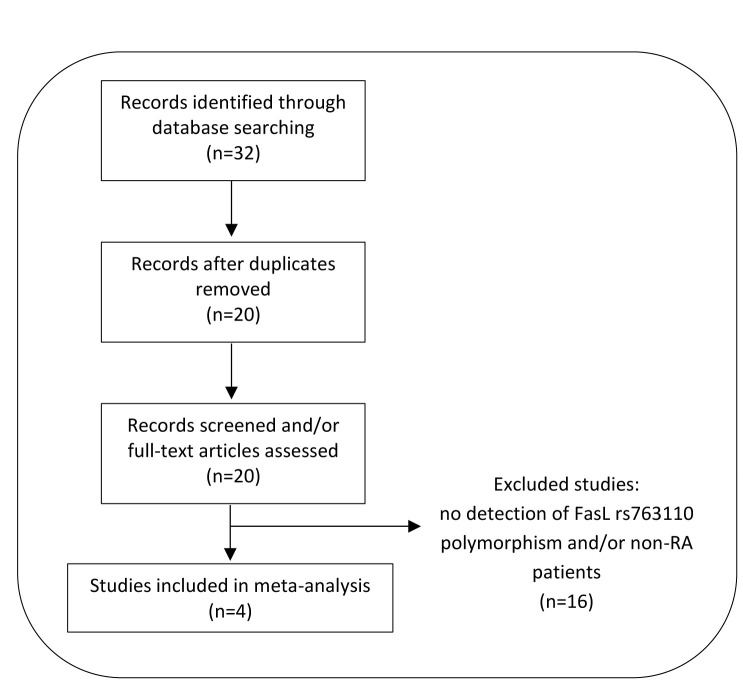
Flow diagram of article selection for meta-analysis.

We searched the Web of Science, Scopus, and PubMed for studies published before May 2020 that investigated the association between the FasL rs763110 polymorphism and susceptibility to RA by using the following keywords: FasL, polymorphism, and rheumatoid arthritis. Duplicate and non-relevant studies were excluded (Figure 1). Except for the four studies included in the meta-analysis conducted by Zhu et al (5,18-20), we found no new eligible studies. After we added the data obtained in our study to the data pool, we conducted an updated meta-analysis using the Review Manager 5 software (Cochrane Collaboration, London, UK). The odds ratio and 95% confidence intervals were calculated using the random effect model.

### Statistical analysis

Clinical parameters and cytokine concentrations are expressed as median with interquartile range (IQR). Differences between groups were tested with the Mann-Whitney or χ^2^ test as indicated for a particular variable, while multiple group comparisons were made by the Kruskal-Wallis test, followed by the Mann-Whitney test with Bonferroni correction. The association between genotypes and RA was analyzed by a free online software SNPStats (http://bioinfo.iconcologia.net/snpstats). A two-tailed p value lower than 0.05 was considered statistically significant. The sample size for two main aims of the study: association of SNPs with RA and TNF-α levels was calculated by setting Type 1 error at 0.05 and Type 2 error at 0.2 (power = 80%).

## RESULTS

### Basic demographic characteristic

There was no significant difference in age (*P* = 0.34, Mann-Whitney test) or sex (*P* = 0.18, χ^2^ test) between the groups. RA patients had high disease activity, with the median DAS 28 of 5.86 (IQR = 4.86-6.69) (Table 1).

**Table 1:** Demographic and clinical characteristics of control participants and rheumatoid arthritis (RA) patients

Characteristic	RA patients	Controls	*P*
N	81	94	-
Age (years)*	65.5 (21-85)	62 (23-91)	0.34
Sex, n (%)^†^			
female	73 (90.12)	77 (81.91)	
male	8 (9.88)	17 (18.09)	0.18
Disease Activity Score-28 score^‡^	5.86 (4.86-6.69)	-	-

*Values are presented as median with range, difference between the groups was tested with the Mann-Whitney test.

†difference between the groups was tested with the χ^2^ test.

‡values are presented as median with interquartile range.

### Patients with RA have a higher concentration of TNF-α in plasma

**Figure 2:**
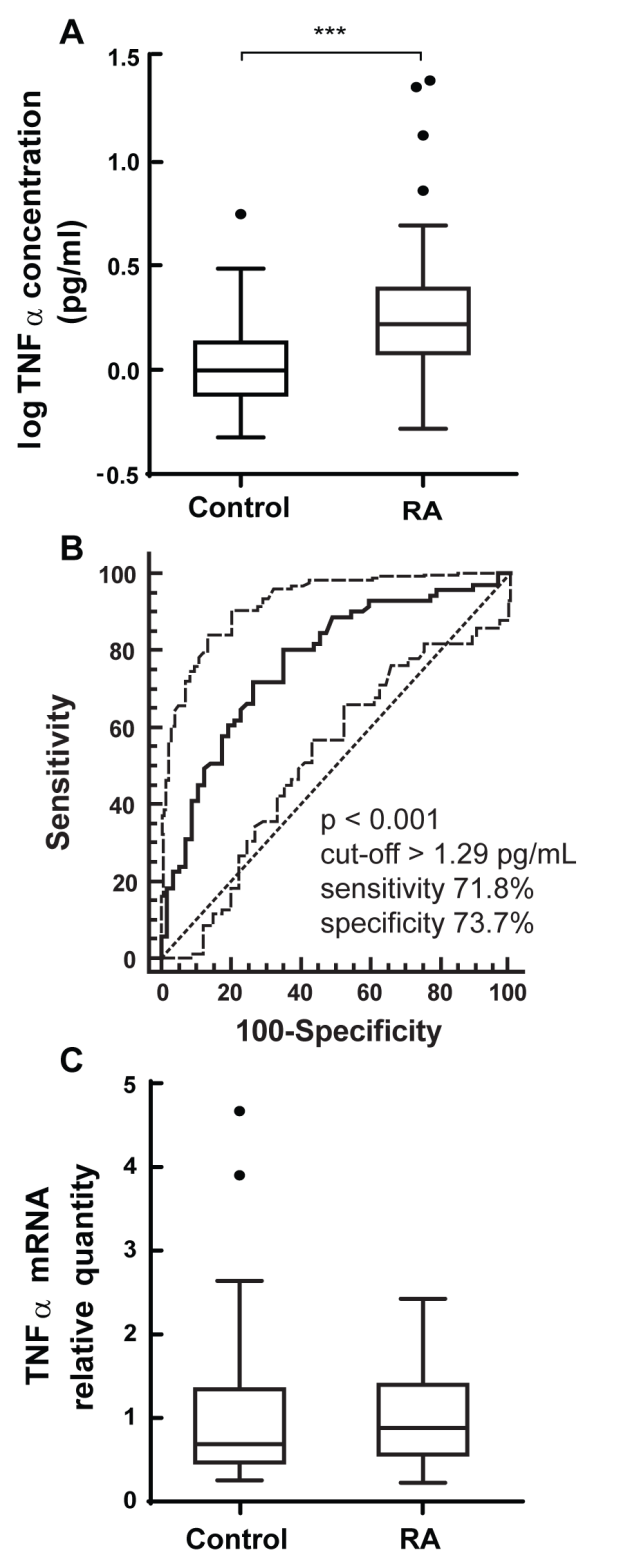
Expression of tumor necrosis factor (TNF)-α in controls and rheumatoid arthritis (RA) patients. (**A**) Concentration of TNF-α in plasma was determined by enzyme-linked immunosorbent assay. (**B**) Receiver operating characteristic (ROC) curve depicts the ability of protein TNF levels to discriminate between controls and RA patients. (**C**) Expression of TNF-α gene in peripheral blood mononuclear cells was determined by quantitative polymerase chain reaction. Boxes indicate the median with interquartile range; bars indicate the minimum and maximum values; and individual points indicate outliers. Comparisons between the groups were made with the Mann-Whitney test. ***P < 0.001

Patients with RA had a significantly higher TNF-α concentration in the plasma (1.65 [1.2-2.42] pg/mL) compared with controls (0.99 [0.77-1.35] pg/mL, *P* < 0.001) (Figure 2A). The analysis of receiver operating characteristic curves revealed that the two groups could be distinguished based on TNF-α levels (area under the curve = 0.77, 95% CI 0.69-0.84, *P* < 0.0001) (Figure 2B). There was no difference in TNF-α gene expression in peripheral blood mononuclear cells (*P* = 0.25, Mann-Whitney test, Figure 2C), which indicates that peripheral blood mononuclear cells are not the source of elevated TNF-α, and might point to the local production of TNF-α in synovial cells. However, further research is warranted to confirm this hypothesis.

### Associations between FasL rs763110 polymorphism and RA

**Figure 3:**
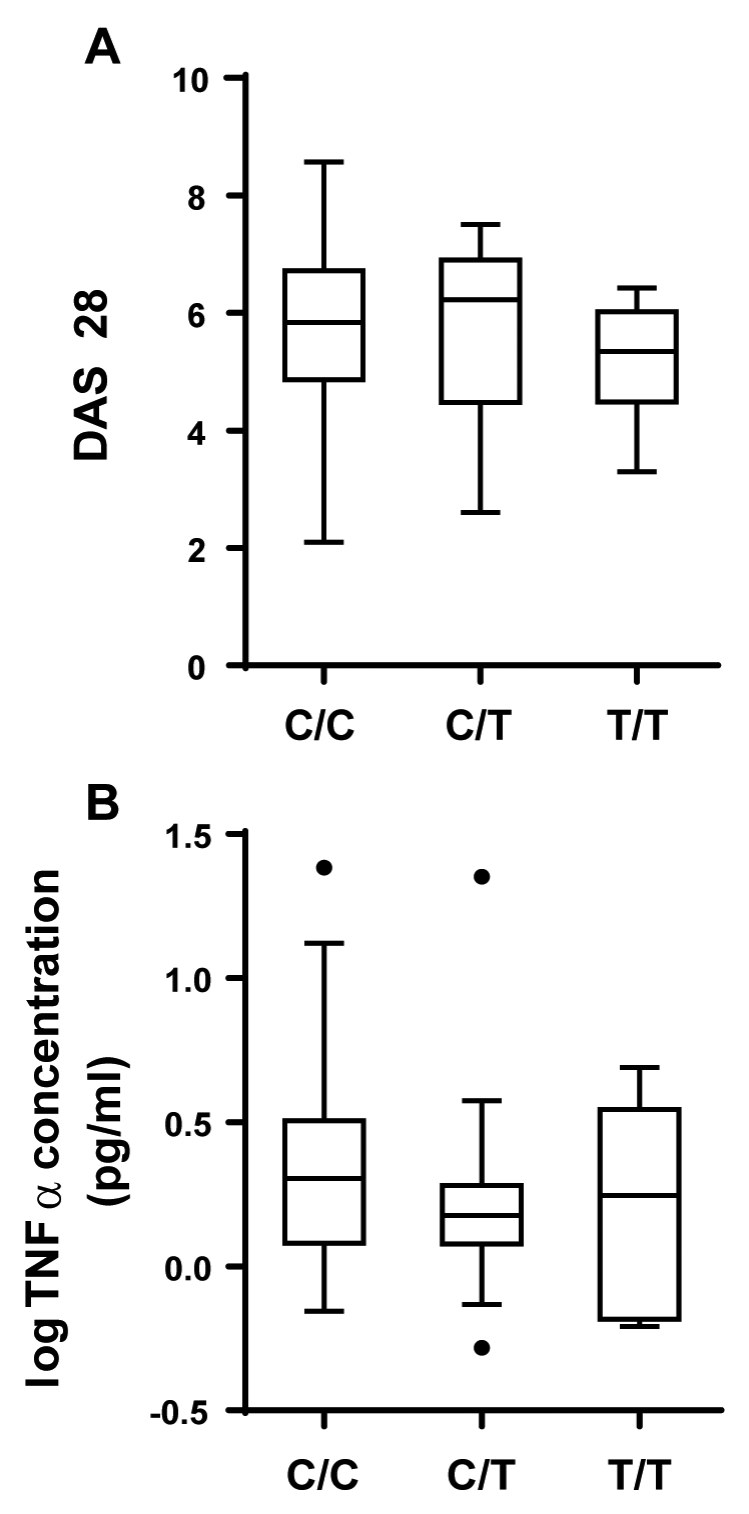
Association between the FasL rs763110 genotype and disease activity indices. (**A**) Disease activity score-28 (DAS 28) was determined during the clinical examination. (**B**) Concentrations of tumor necrosis factor-α in plasma were determined by enzyme-linked immunosorbent assay. Boxes indicate median with interquartile range; bars indicate the minimum and maximum values; and individual points indicate outliers. Comparisons between groups were made with the Kruskal-Wallis test.

The distribution of FasL rs763110 genotypes was in Hardy-Weinberg equilibrium in both the case (*P* = 0.81) and control (*P* = 0.13) group. The frequency of the C allele in all participants was 64% (62% in controls and 65% in RA group, Table 2). The presence of the T allele was not significantly associated with the occurrence of RA (OR with 95% CI = 0.87 [0.56-1.35], *P* = 0.54). The association between the FasL rs763110 polymorphism and RA was further tested in codominant, dominant, recessive, overdominant, and log additive model (Table 3). In all the tested models, we found no significant association (*P* = 0.36, 0.94, 0.19, 0.31, and 0.55 respectively). The RA patients with various rs763110 genotypes did not significantly differ in DAS 28 score (5.84 [4.87-6.71] for CC, 6.23 [4.57-6.86] for CT, and 5.35 [4.88-5.88] for TT, *P* = 0.59, Kruskal-Wallis test) (Figure 3A) or TNF-α concentration (2.02 [1.21-3.08] for CC, 1.5 [1.19-1.9] for CT, and 1.76 [0.02-3.1] for TT, *P* = 0.24, Kruskal-Wallis test, values in pg/mL) (Figure 3B).

**Table 2:** Genotype and allele frequencies in control participants and rheumatoid arthritis (RA) patients

	No. (%) of	
Genotype/allele	RA patients	controls
CC	34 (42)	40 (43)
CT	38 (47)	37 (39)
TT	9 (11)	17 (18)
C	106 (65)	117 (62)
T	56 (35)	71 (38)
Hardy-Weinberg equilibrium	0.81	0.13

**Table 3:** Association between the FasL rs763110 genotype and rheumatoid arthritis (RA) in various models

		No. (%) of			
Model	Genotype/allele	RA patients	controls	Odds ratio	*P*
Allelic association	C	106 (65)	117 (62)	1.00	0.54
	T	56 (35)	71 (38)	0.87 (0.56-1.35)	
Codominant	CC	34 (42)	40 (42.5)	1.00	0.36
	CT	38 (46.9)	37 (39.4)	1.21 (0.63-2.3)	
	TT	9 (11.1)	17 (18.1)	0.62 (0.25-1.58)	
Dominant	CC	34 (42)	40 (42.5)	1.00	0.94
	CT + TT	47 (58)	54 (57.5)	1.02 (0.56-1.87)	
Recessive	CC + CT	72 (88.9)	77 (81.9)	1.00	0.19
	TT	9 (11.1)	17 (18.1)	0.57 (0.24-1.35)	
Overdominant	CC+TT	43 (53.1)	57 (60.6)	1.00	0.31
	CT	38 (46.9)	37 (39.4)	1.36 (0.75-2.48)	
Log-additive	-			0.88 (0.58-1.34)	0.55

### Updated meta-analysis

**Figure 4:**
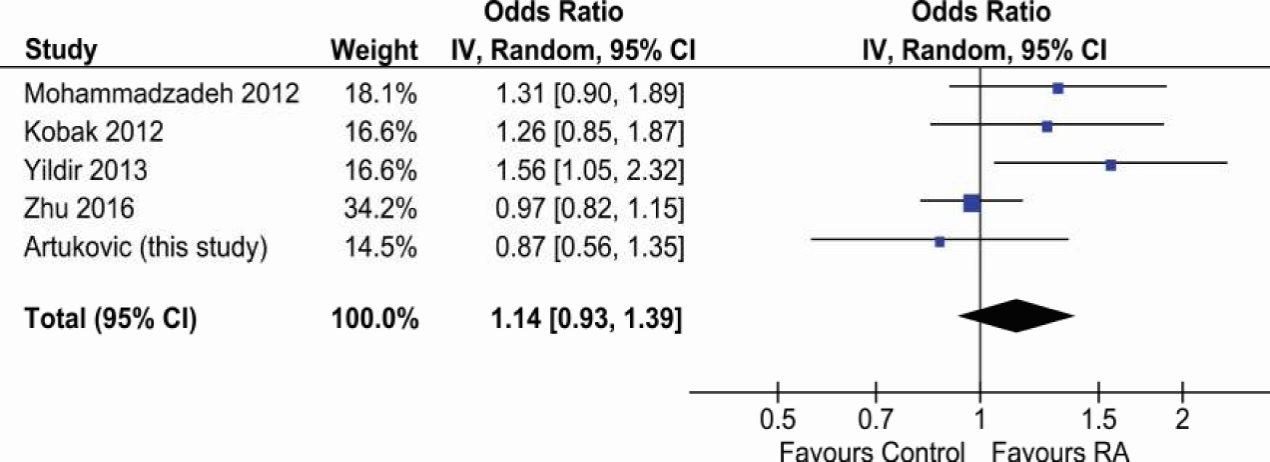
Forest plot for the association between FasL rs763110 allele and rheumatoid arthritis (RA) in all studies. Squares and lines represent the odds ratio with 95% confidence intervals (CI) of individual studies, while the surface of the square indicates the weight of the study. The total effect was calculated by a random model and is presented by a diamond showing the odds ratio with 95% CI.

**Figure 5:**
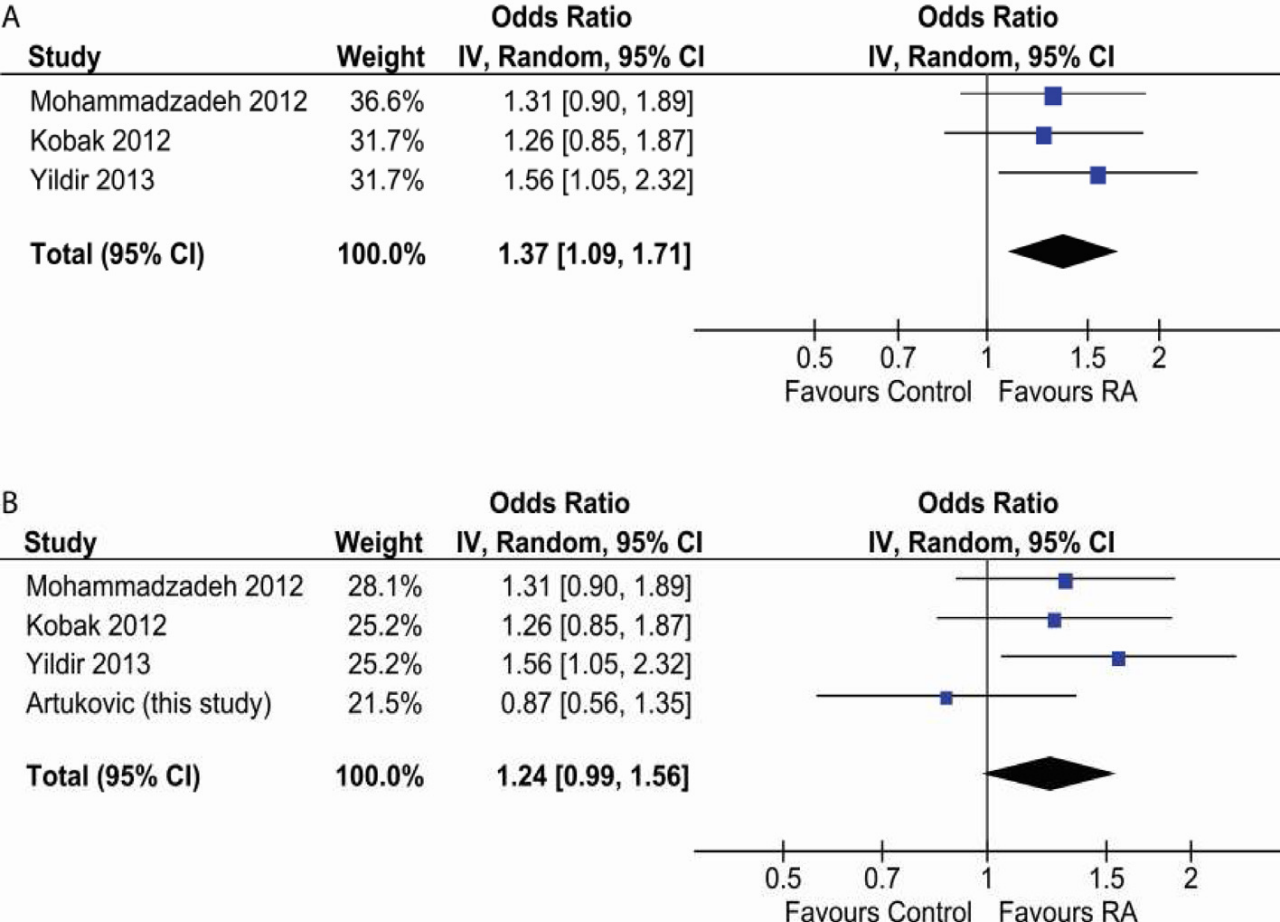
Forest plot for the association between FasL rs763110 allele and rheumatoid arthritis (RA) in the Western Eurasian subgroup. (**A**) Without this study. (**B**) With this study. Squares and lines represent the odds ratio with 95% confidence intervals of individual studies, the surface of the square indicates the weight of the study. The total effect was calculated by a random model and is presented by a diamond showing the odds ratio with 95% confidence intervals.

Our study results were added to the results of four eligible previous studies in a meta-analysis (Table 4). No significant association was established in any of the investigated models when the effect of all studies was analyzed (Table 5 and Figure 4). In a previously conducted stratification analysis, Zhu et al reported a significant association in the Western Eurasian population in three tested models: T vs C (*P* = 0.006), TT+CT vs CC (*P* = 0.014), and TT vs CC (*P* = 0.01). Therefore, we added our study participants to the stratification analysis as Western Eurasians, and conducted an updated meta-analysis of these models. The significant association was abolished in T vs C (OR with 95% confidence intervals, 1.24 [0.99-1.56], *P* = 0.063) and TT vs CC model (1.45 [0.84-2.49], *P* = 0.184), while TT+CT vs CC model still showed a significant association (1.39 [1.03-1.88], *P* = 0.029) (Figure 5, Table 6).

**Table 4:** Overview of the studies included in the meta-analysis

Study	Country	Genotype frequencies						Allele frequencies				Hardy-Weinberg equilibrium
		controls			rheumatoid arthritis patients			controls		rheumatoid arthritis patients		
		CC	CT	TT	CC	CT	TT	C	T	C	T	
Mohammadzadeh, 2012	Iran	43	49	20	33	63	24	135	89	129	111	0.36
Kobak,2012	Turkey	33	40	23	30	40	31	106	86	100	102	0.12
Yildir, 2013	Turkey	31	54	14	20	55	25	116	82	95	105	0.22
Zhu, 2016	China	453	317	51	331	228	34	1223	419	890	296	0.65
Artukovic (this study)	Croatia	40	37	17	34	38	9	117	71	106	56	0.33

**Table 5:** Meta-analysis of association between the FasL rs763110 genotype and rheumatoid arthritis (RA) occurrence

Model	Odds ratio	*P*
allelic association (T vs C)	1.14 (0.93-1.39)	0.21
dominant (CT + TT vs CC)	1.19 (0.92-1.54)	0.18
recessive (TT vs CT+CT)	1.12 (0.79-1.61)	0.52
overdominant (CT vs CC + TT)	1.06 (0.89-1.25)	0.54
TT vs CC	1.26 (0.81-1.97)	0.3

**Table 6:** Comparison of meta-analysis results in Western Eurasian subgroup with and without the inclusion of this study

Included studies	Western Eurasians without this study		Western Eurasians with this study	
Model	odds ratio	*P*	odds ratio	*P*
allelic association (T vs C)	1.37 (1.09-1.71)	0.006	1.24 (0.99-1.56)	0.06
dominant (CT + TT vs CC)	1.54 (1.09-2.18)	0.01	1.39 (1.03-1.88)	0.03
TT vs CC	1.79 (1.14-2.79)	0.01	1.45 (0.84-2.49)	0.18

## DISCUSSION

We found no association between the FasL rs763110 polymorphism and rheumatoid arthritis in the Croatian population. To the best of our knowledge, this is the first such study conducted in Europeans. As polymorphisms of the genes involved in the regulation of apoptosis might alter apoptotic pathways, they are often found to be associated with RA susceptibility and progression. For example, a significant association between the genotype and RA susceptibility was reported for Fas, FasL, survivin, or programmed cell death 1 gene polymorphisms (21,22). However, because of ethnic differences, reported associations cannot be directly extended to the global population.

The frequency of a minor allele (T) in all participants was 36%, and the distribution of genotypes was in Hardy-Weinberg equilibrium, which is similar to other studied populations. Although recent studies suggested that FasL (rs763110) polymorphism was associated with RA in Western Eurasians, we found no evidence of such association in Croatians (p value was between 0.19 and 0.94 depending on the model of analysis). Similar results were reported by Zhu et al in the Chinese population. Our updated meta-analysis of all eligible published studies confirmed a lack of association in the overall population. Furthermore, when the results of this study were added to Western Eurasian population subgroup in the updated meta-analysis, the findings of the stratification meta-analysis conducted by Zhu (5) that FasL (rs763110) polymorphism was associated with RA in Western Eurasians were abolished (for allelic and TT vs CC models) or weakened (for the dominant model).

The levels of TNF-α, which is one of the pivotal mediators in the pathogenesis of RA, were, expectedly, elevated in RA patients. Interestingly, this was not reflected in the mRNA from peripheral mononuclear cells. This indicates that TNF-α might be primarily produced locally, which agrees with the previous findings that it is produced by synovial cells – mainly monocytes and macrophages, but also B cells, T cells, and fibroblasts (23). However, we did not confirm this in synovial samples and, therefore, this conclusion should be taken with caution.

Receptors for TNF-α (TNFR1 and TNFR2) and FasL (Fas, also known as CD95 or TNFRSF6) are both members of the TNF receptor superfamily. Various *in vitro* and *in vivo* studies have suggested complex crosstalk between their signaling pathways. Furthermore, it was found that TNF-α and FasL affected each other’s activity and expression (8,24). Therefore, we hypothesized that the rs763110 genotype might be associated with TNF-α concentration in plasma, which had not been previously investigated. However, the differences in TNF-α levels between RA patients of various FasL rs763110 genotypes were not significant.

Finally, we also tested the possibility that the FasL rs763110 genotype might be associated with the index of RA activity (DAS 28 score). We found no significant association between the genotype and DAS 28. This is in accordance with previous studies by Kobak et al and Yildir et al, as both of these studies found no such association (18,19). However, it should be noted that this still does not completely rule out the possibility that FasL rs763110 affects disease activity, as an ideal comparison would be that of patients with persistently active disease (like those in our study group) and those achieving (longer) remission periods.

In summary, our data suggest that the association between FasL rs763110 polymorphism and RA susceptibility in Western Eurasians observed in previous studies might be overestimated and should be limited to the population of Southwestern Asia until further investigations are performed. Our results support the conclusions of previous studies that different genotypes were not associated with RA activity as determined by DAS 28 score. Finally, we found no significant association between the FasL rs763110 genotypes and TNF-α concentration in the plasma of RA patients.
